# Variational Bayesian Compressive Sensing with Equivalent Source Modeling for Sound Field Reconstruction

**DOI:** 10.3390/s26041145

**Published:** 2026-02-10

**Authors:** Yue Xiao, Zhepu Chen, Haiyang Zhang, Chengping Zhong

**Affiliations:** 1 Jiangxi Provincial Key Laboratory of Precision Drive and Equipment, Jiangxi University of Water Resources and Electric Power, Nanchang 330099, China; 18232163533@163.com (Z.C.); 19942180838@163.com (H.Z.); 2 Jiangxi Key Laboratory of Intelligent Connected Vehicle and Powertrain System, Jiangling Motors Corporation Limited, Nanchang 330020, China; czhong4@ford.com

**Keywords:** near-field acoustic holography, equivalent source method, compressive sensing, variational Bayesian, Kullback–Leibler divergence

## Abstract

While conventional Bayesian compressive sensing exploits signal sparsity for accurate sound field reconstruction from under-sampled measurements, its practicality is limited by high computational complexity and slow convergence. To address these limitations, this paper proposes a variational Bayesian compressive sensing framework integrated with equivalent source modeling for sound field reconstruction. The approach first establishes a sparse representation of the sound field using the equivalent source method, and then assigns hierarchical prior distributions to the equivalent source strengths and the noise precision within this Bayesian model. Mean-field variational inference is adopted to derive an analytically tractable approximation to the true posterior distribution by minimizing the Kullback–Leibler divergence, thus enabling efficient estimation of the equivalent source strengths and subsequent high-accuracy sound field reconstruction. This proposed method retains the desirable statistical advantages of Bayesian modeling while enhancing computational efficiency. Numerical simulations and experiments validate that the proposed method achieves superior reconstruction accuracy compared with conventional Bayesian compressive sensing and orthogonal matching pursuit algorithm, with significantly reduced computational burden and enhanced robustness in low signal-to-noise ratio scenarios.

## 1. Introduction

Near-field acoustic holography (NAH) is a powerful technique for noise source identification and characterization, reconstructing sound fields from limited acoustic measurements, with widespread applications in noise control and fault diagnosis [1,2,3,4,5]. However, traditional NAH relies on the Nyquist sampling theorem [6], where spatial resolution is constrained by sampling density, leading to high measurement costs and limited engineering applications. In recent years, compressive sensing (CS) theory [7,8,9] has provided an innovative approach to break through the limitations of the Nyquist sampling theorem. This theory leverages the sparse characteristics of signals, stating that if a signal is inherently sparse or sparse in some transform domain, the original high-dimensional signal can be linearly projected into a lower-dimensional space using a measurement matrix uncorrelated with the sparse basis. Within the CS framework, only a small number of measurements—far fewer than those required by the Nyquist sampling rate—are needed to accurately reconstruct the original signal with high probability. Introducing CS theory into NAH technology exploits the sparse characteristics of sound field signals to achieve under-sampled measurements, thereby effectively overcoming the limitations imposed by the Nyquist sampling law [10,11,12]. CS-based NAH methods enable sound field reconstruction with higher spatial resolution while reducing the number of microphone array points, enhancing the potential for practical engineering applications.

To balance reconstruction accuracy and computational efficiency, various compressive sensing reconstruction algorithms have been developed, primarily including greedy algorithms [13,14,15], convex optimization algorithms [16,17,18], and Bayesian compressive sensing (BCS) [19,20,21]. Greedy algorithms achieve signal reconstruction by directly solving the *l*_0_-norm minimization problem. Their core principle involves iteratively determining the support set of the sparse signal based on specific selection criteria. These algorithms feature low computational complexity and fast operation speeds. However, they often fail to guarantee globally optimal solutions, and are prone to local optima. Crucially, their accuracy deteriorates significantly under conditions of high column coherence in the sensing matrix or low signal-to-noise ratio (SNR). Convex optimization algorithms utilize the *l*_1_-norm as a convex relaxation of the *l*_0_-norm, transforming the originally intractable non-convex optimization problem into a convex one for solution. When the sensing matrix satisfies the restricted isometry property (RIP) condition [22], this algorithm can find the global optimal solution with a small number of measurements while maintaining high reconstruction accuracy and noise resistance. However, it suffers from high computational complexity and slow execution. Furthermore, its theoretical performance guarantees are contingent upon the sensing matrix satisfying the RIP condition. Sparse Bayesian algorithms frame the signal reconstruction problem within a probabilistic framework. They achieve signal recovery by designing sparse prior distributions for the unknown signal and employing Bayesian estimation for maximum a posteriori (MAP) estimation. These algorithms can adaptively estimate parameters such as signal sparsity and noise level, and typically yield globally optimal solutions with high sparsity. Given that Bayesian estimation also provides the capability for uncertainty quantification and adaptively estimates signal sparsity, it effectively avoids the greedy algorithms’ reliance on prior knowledge of sparsity and the stringent requirement for the RIP condition in convex optimization algorithms. Consequently, it has been widely adopted by scholars in various fields. Lin et al. [23] proposed a method combining block sparse Bayesian learning (BSBL) with a grid refinement strategy by leveraging the structural information of source signals, including spatial sparsity and temporal correlation of the sources, and achieved high-performance direction of arrival (DOA) estimation. Park et al. [24] proposed a two-dimensional off-grid beamforming method based on block sparse compressed sensing. By jointly treating off-grid compensation terms and discrete grid terms as a block sparse signal, this method exhibited high-resolution and high-precision estimation performance in both simulation and experimental data, effectively mitigating the limitations of grid-based beamforming. In terms of broadband mode extraction in shallow-water acoustics, Niu et al. [25] developed a block sparse Bayesian learning method leveraging data from a vertical array. The Bayesian framework adaptively estimates modal wavenumbers and depth functions, thereby effectively resolving broadband mode separation without requiring prior knowledge of mode orders, which enhances its practicality in complex marine acoustic environments. Das [26] proposed a unitary transformation-based real-valued sparse Bayesian learning algorithm combined with an off-grid model for wideband DOA estimation. This method achieves automatic DOA estimation, significantly reduces computational complexity, and was validated in the HF97 ocean experiment, demonstrating high resolution and robustness against noise and correlated signals. For acoustic holography, Yu et al. [27] presented an SBL-based approach that achieves high-precision sound source identification and reconstruction from a single measurement plane. A key advantage of this method is its ability to adaptively estimate source parameters without manual regularization, simplifying the operation process while ensuring accuracy. Equivalent source methods have also been upgraded with block sparse Bayesian learning. Xiao et al. [28] integrated Bayesian compressive sensing with the equivalent source method, adaptively solving for the sparse coefficients of sound sources via Bayesian estimation. This method achieves high-accuracy full-frequency-band sound field reconstruction even with sparse sampling and a low signal-to-noise ratio, thereby enhancing its robustness and practical applicability. Bi et al. [29] proposed a corresponding method by constructing a block-sparse equivalent source model and imposing structured priors; it can adapt to different sound source types merely by adjusting the block size, thereby realizing the reconstruction of radiated sound fields for various sources. Zhang et al. [30] further enhanced this approach with a hyperparameter-coupled prior, employing a hierarchical model to autonomously determine regularization parameters and achieve robust reconstruction with superior noise immunity. Most recently, Pan et al. [31] proposed a sound source identification method that integrates a multipole transfer matrix model with sparse Bayesian learning. By combining physical mechanisms with a data-driven framework, this method enables efficient sound field modeling and precise source localization and strength reconstruction in enclosed spaces, expanding the application scope of SBL-based methods to confined acoustic environments.

Despite significant advances, the aforementioned sparse-Bayesian-learning-based approaches still suffer from intrinsic limitations. Relying on iterative point estimation or type-II maximum likelihood for hyperparameter inference often incurs prohibitive computational cost, especially in large-scale problems. Most existing methods struggle to fully quantify the uncertainty inherent in reconstruction, merely delivering point estimates devoid of reliable confidence intervals. Manual tuning of critical parameters, such as block size or pruning thresholds, introduces subjectivity that may compromise result robustness. Assumptions about specific prior distributions can restrict the model’s flexibility in capturing complex source characteristics. The integration of physical models with the learning framework tends to be shallow, lacking a Bayesian mechanism that effectively balances data fidelity and physical constraints.

To address this issue, researchers have turned to computational approximations to reduce complexity. In recent years, variational Bayesian (VB) inference [32] has gained significant traction as a scalable deterministic approximation method particularly suited to high-dimensional problems, offering enhanced computational efficiency. The core idea underpinning VB is to replace the intractable complex posterior distribution with a simpler variational distribution of a tractable form, transforming this approximation task into an optimization process that minimizes the Kullback–Leibler (KL) divergence. This transformation ingeniously incorporates the complex integration problem into an efficiently solvable optimization framework, providing a feasible technical pathway for practical applications. Recently, this method has demonstrated significant advantages in multiple signal processing fields. For instance, Cui et al. [33] established a VB inference framework for DOA estimation, effectively leveraging the temporal correlation of signals to significantly improve convergence speed and estimation accuracy while avoiding complex matrix operations. Liu et al. [34] introduced a subspace-constrained VB method into structured compressed sensing reconstruction; through a parameter self-adaptive updating mechanism, it achieved reconstruction performance comparable or superior to mainstream methods at a lower iterative cost. Li et al. [35], addressing joint localization in smart surface-assisted systems, utilized sparsity in the angular domain and realized alternating estimation of location and channel parameters via VB learning, effectively avoiding high-dimensional global search. Wan et al. [36] further extended VB to multi-antenna detection, constructing an inversion-free inference network that maintained robust performance even in environments with noise uncertainty. Collectively, these studies demonstrate that VB, by recasting complex inference as tractable optimization, achieves a favorable balance: it dramatically reduces computational complexity and runtime while maintaining robust estimation performance across diverse complex scenarios. This makes VB a compelling solution for high-dimensional signal processing, effectively balancing efficiency and performance.

Motivated by the computational efficiency of VB, this paper integrates variational Bayesian inference with compressive sensing, proposing a variational Bayesian compressive sensing (VBCS) algorithm for sound field reconstruction. This algorithm utilizes near-field acoustic holography based on the equivalent source method to achieve sound field reconstruction by solving for the sparse coefficient vector of equivalent source strengths within the compressive sensing framework. Diverging from BCS, which attempts direct computation of the intractable true posterior distribution, the proposed VBCS seeks a tractable variational distribution as an approximation. This approach effectively transforms the intractable problem of marginal likelihood integration in BCS into an efficiently solvable optimization problem by minimizing the Kullback–Leibler divergence between the variational and true posterior distributions. This objective avoids the intractable integration terms, allowing the inference process to bypass direct high-dimensional integration and enabling effective inference of the unknown parameters. Consequently, VBCS achieves significantly reduced computational complexity while preserving the theoretical advantages of BCS, notably implicit regularization and robustness to over-fitting. Moreover, its applicability extends to scenarios with unknown noise variance, enhancing its practical utility and adaptability in engineering applications.

The structure of this paper is organized as follows: First, a brief introduction is provided on the application of near-field acoustic holography and compressive sensing in the field of sound source identification. Section 2 elaborates on the fundamental theories of the equivalent source method and variational Bayesian compressive sensing. Section 3 validates the performance of the proposed method through numerical simulations and comparisons with traditional Bayesian compressive sensing and orthogonal matching pursuit (OMP). Section 4 further verifies the effectiveness of the proposed method through experimental studies. Subsequently, Section 5 discusses the limitations of the proposed algorithm and outlines directions for future work. Finally, Section 6 concludes the paper with a summary of the key findings.

## 2. Theory

### 2.1. ESM in the CS Framework

The fundamental principle of near-field acoustic holography based on the equivalent source method ESM is to represent the sound field radiated by a source of arbitrary geometry by the superposition of fields generated by a set of equivalent sources distributed on a virtual source plane. Using the ESM, a system of equations relating the measured sound pressures to the equivalent source strengths is established. These strengths are then determined by matching the boundary conditions on the source or hologram surface. A key advantage of this method is that it does not involve Fourier transforms in the wavenumber domain, thereby fundamentally avoiding wraparound errors and singular integrals. Consequently, the ESM-based NAH is characterized by its simplicity, high computational efficiency, and adaptability to sound sources of arbitrary shape.

**Figure 1:**
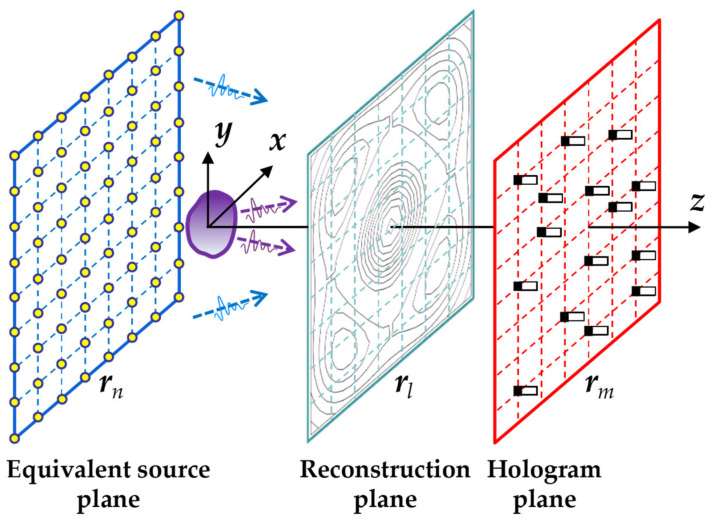
Schematic diagram of the equivalent source method.

The schematic diagram of the basic principle of the equivalent source method is shown in Figure 1. The actual sound field is approximated by the superposition of waves radiated from a set of discrete equivalent sources. The strengths of these sources are recovered from the sound pressures measured on the hologram plane, enabling the prediction and reconstruction of the entire acoustic field. Consider *N* equivalent sources distributed on the equivalent source plane and an array of *M* microphones on the measurement plane. The acoustic transfer relationship between the sound pressure on the hologram plane and the equivalent source strengths can be expressed as

(1) $$P_{h}=T_{\mathrm{sh}}Q+\varepsilon$$

where ***P***_h_ denotes the complex sound pressure vector at the *M* measurement points on the hologram plane, ***Q*** represents the strengths vector of the *N* equivalent sources, ***ε*** is the noise error vector. $T_{\mathrm{sh}}(m,n)=\exp(ik|r_{m}-r_{n}|)/\left(4\pi |r_{m}-r_{n}|\right)$ is the transfer matrix between the *m*-th measurement point ***r****_m_* and the *n*-th equivalent source point ***r****_n_*, and *k* is the wave number.

Within the compressive sensing framework, the equivalent source strengths can be sparsely represented via a sparse basis matrix

(2) Q=Φw

where ***Φ*** is the sparse basis matrix for the equivalent source strength vector ***Q***, and ***w*** is the sparse coefficient vector of the equivalent source strengths with only *K* (*K* ≪ *N*) non-zero entries.

Thus, the reconstructed sound pressure can be expressed in terms of the equivalent source strengths as

(3) $$P_{r}=T_{sr}Q$$

where ***P***_r_ is the sound pressure vector on the reconstruction plane, $T_{\mathrm{sr}}(l,n)=\exp(ik|r_{l}-r_{n}|)/\left(4\pi |r_{l}-r_{n}|\right)$ is the transfer matrix between the *l*-th reconstruction point ***r****_l_* and the *n*-th equivalent source point ***r****_n_*.

To realize sparse reconstruction in the compressive sensing framework, the equivalent source method ESM requires a physically meaningful and mathematically rigorous sparse basis. In this study, the right singular matrix ***V*** of the transfer matrix ***T***_sr_ is strictly selected as the sparse basis, which is derived from the singular value decomposition (SVD) of ***T***_sr_ = ***USV***^H^, where ***U*** and ***V*** are the left and right unitary matrices, respectively, and ***S*** is a diagonal matrix composed of positive singular values. The superscript “H” denotes the conjugate transpose.

Mathematically, the left singular vector matrix ***U*** constitutes an orthogonal basis for the reconstruction space, with dimensions matching the number of reconstruction points. This basis ***U*** naturally represents the modal space of reconstructed sound pressures, making ***U*** appropriate for decomposing ***P***_r_ but not equivalent source strengths. Conversely, the right singular vector matrix ***V*** spans the source space, dimensioned by the number of equivalent sources. In ESM, the transfer matrix ***T***_sr_ maps source strengths to reconstructed pressures, and its right singular vectors ***V*** directly constitute the basis for source-strength space. This aligns with the CS objective: achieving sparsity in source-strength representation. Thus, ***U*** compresses reconstructed pressure data, whereas ***V*** enables sparse control of equivalent sources.

Physically, the right singular vector matrix ***V*** corresponds to acoustic radiation modes. As Borgiotti et al. [37] and Photiadis [38] showed, these modes are ordered in descending radiation efficiency, so the first few capture most of the acoustic energy and naturally yield a sparse representation. For spatially extended sources, ***V*** describes the radiation pattern without extra geometric assumptions. Among competing sparse bases, ***V*** represents the sound field with the fewest functions, satisfying the CS incoherence requirement. Unlike the compressive ESM (C-ESM) [39] and the real-valued modal basis used in compressed modal ESM (CMESM) [40], ***V*** retains more phase information and exhibits superior high-frequency performance. Studies [41,42,43,44] have demonstrated that ***V*** significantly enhances both the sparsity of the solution and the accuracy of reconstruction. Adopting the right singular matrix ***V*** as the sparse basis for ESM within the compressive sensing framework provides three key advantages in sound field reconstruction. First, its adaptivity is manifested in the direct derivation of ***V*** from the transfer matrix, ensuring automatic conformity to any geometry or boundary condition. Second, it provides energy compaction because the SVD ordering inherently concentrates the acoustic energy into the fewest possible components. Third, the framework ensures stability, as the SVD-based representation functions as a built-in regularizer, thereby mitigating the ill-posedness introduced by measurement noise.

Based on the above, the right singular matrix ***V*** can be selected as the sparse basis matrix, as it has been demonstrated to achieve high reconstruction accuracy, i.e., ***Φ*** = ***V***.

Substituting Equation (2) into Equation (1), the measured sound pressure on the hologram plane can be expressed as

(4) $$P_{h}=T_{\mathrm{sh}}Vw+\varepsilon =Hw+\varepsilon$$

where $H=T_{\mathrm{sh}}V$ is the sensing matrix.

When the number of measurement points *M* is less than the number of equivalent sources *N*, the sound field reconstruction problem becomes ill-posed, and it is impossible to obtain a stable and accurate reconstruction signal. According to compressive sensing theory, the sparse coefficient vector of the equivalent source strengths in Equation (4) can be solved by minimizing the *l*_1_-norm

(5) $$\arg\underset{w}{\min}\left({\left\|P_{h}-Hw\right\|}_{2}^{2}+\lambda {\left\|w\right\|}_{1}\right)$$

The core of equivalent source-based sound field reconstruction using compressive sensing lies in utilizing the sound pressure measurements ***P***_h_ on the hologram plane to solve for the sparse coefficient vector of the equivalent source strengths ***w***.

### 2.2. Sound Field Reconstruction Based on VBCS

Although the traditional Bayesian compressive sensing algorithm can directly solve the sparse solution of Equation (5), its real-valued probability model is not suitable for complex sound pressure signals and cannot separately process the real and imaginary components. Therefore, it is necessary to decompose Equation (4) into real and imaginary parts and reformulate it as

(6) P=CW+n

with P=RePhImPh, C=ReHImH−ImHReH, W=RewImw, n=ReεImε, $P\in R^{2M}$, $C\in R^{2M\times 2N}$, $W\in R^{2N}$, $n\in R^{2M}$, where Re(·) and Im(·) denote the real and imaginary parts of taking the corresponding quantities, respectively.

According to Bayesian estimation theory, assuming both the real and imaginary parts of the noise error ***n*** follow a zero-mean Gaussian distribution with a precision of *β*, the probability density of ***P*** can be expressed as

(7) $$P\left(P|W,\beta \right)={\left(2\pi \beta \right)}^{-2M}\exp\left(-\frac{1}{2\beta }{\left(P-CW\right)}^{T}\left(P-CW\right)\right)$$

The Bayesian compressive sensing algorithm controls the sparsity of the sparse coefficient vector ***W*** of the equivalent source strengths by setting its hyperparameters ***α***, where each element *W_i_* follows a zero-mean Gaussian prior distribution with variance $\alpha_{i}^{-1}$

(8) $$P\left(W|\alpha \right)=\prod_{i=1}^{2N}N\left(W_{i}|0,\alpha_{i}^{-1}\right)$$

where $\alpha =\left[\alpha_{1},\alpha_{2},\ldots ,\alpha_{2N}\right]$ determines the prior distribution of the sparse vector ***W*** for the equivalent source strengths. Obviously, when *α_i_* approaches positive infinity, the corresponding *W_i_* tends toward zero.

Furthermore, a new layer of distribution parameters is introduced for the hyperparameter ***α***, enabling adaptive learning through maximization of the posterior probability. Within the hierarchical Bayesian framework, and to exploit the conjugate prior property of the precision parameter in Gaussian distributions, a Gamma hyperprior is placed on the hyperparameter ***α*** as

(9) $$P\left(\alpha \right)=\prod_{i=1}^{2N}\Gamma \left(\alpha_{i}|a,b\right)=\prod_{i=1}^{2N}\Gamma (a{)}^{-1}b^{a}\alpha_{i}^{a-1}e^{-b\alpha_{i}}$$

where $\Gamma (a)=\int_{0}^{\infty }t^{a-1}e^{-t}dt$ is the Gamma function, and *a* and *b* are the shape parameter and the rate parameter, respectively.

Similarly, a Gamma hyperprior is placed on the noise precision *β* as

(10) $$P\left(\beta \right)=\Gamma (\beta |c,d)=\Gamma {\left(c\right)}^{-1}d^{c}\beta^{c-1}e^{-d\beta }$$

where Γ(β|c,d) denotes the Gamma distribution, *c* is the shape parameter, and *d* is the scale parameter.

According to Bayesian theory, solving for the sparse coefficient vector ***W*** of the equivalent source strength can be transformed into the problem of maximizing the joint posterior probability:

(11) $$P\left(W,\alpha ,\beta |P\right)=\frac{P\left(P|W,\alpha ,\beta \right)P\left(W,\alpha ,\beta \right)}{P\left(P\right)}$$

While the Bayesian compressive sensing algorithm can accomplish sound field reconstruction, its computational process requires integration over all parameters when calculating the joint posterior probability. This renders the integrals complex and intractable due to the high-dimensional nature of the problem, thereby compromising the computational efficiency of the algorithm. Variational Bayesian inference is introduced to the Bayesian compressive sensing framework to alleviate its computational burden and enhance operational speed.

Specifically, first define the parameter set ***Ω*** composed of unknown parameters to be estimated as

(12) $$\Omega =\left\{W;\alpha ;\beta \right\}$$

The marginal probability density of the observed data ***P*** is

(13) $$P\left(P\right)=\frac{P\left(P,\Omega \right)}{P\left(\Omega |P\right)}$$

Based on variational inference theory, introduce a variational distribution *q*(***Ω***) concerning the parameter set into Equation (13) as

(14) $$P\left(P\right)=\frac{P\left(P,\Omega \right)q\left(\Omega \right)}{P\left(\Omega |P\right)q\left(\Omega \right)}$$

Taking the logarithm of both sides of Equation (14) yields

(15) $$\ln\left(P\left(P\right)\right)=\ln\frac{P\left(P,\Omega \right)}{q\left(\Omega \right)}-\ln\frac{P\left(\Omega |P\right)}{q\left(\Omega \right)}$$

Set $∭q\left(\Omega \right)d\Omega =1$, Equation (15) can be transformed into

(16) $$\ln\left(P\left(P\right)\right)=L\left(q\left(\Omega \right)\right)+\mathrm{KL}\left(q\left(\Omega \right)||P\left(\Omega |P\right)\right)$$

where

(17) $$L\left(q\left(\Omega \right)\right)=∭q\left(\Omega \right)\ln\frac{P\left(P,\Omega \right)}{q\left(\Omega \right)}d\Omega$$

(18) $$\mathrm{KL}\left(q\left(\Omega \right)||P\left(\Omega |P\right)\right)=-∭q\left(\Omega \right)\ln\frac{P\left(\Omega |P\right)}{q\left(\Omega \right)}d\Omega$$

In the above, $\mathrm{KL}\left(q\left(\Omega \right)||P\left(\Omega |P\right)\right)$ is called the Kullback–Leibler (KL) divergence, indicating the approximation degree between the variational distribution *q*(***Ω***) and the posterior distribution $P\left(\Omega |P\right)$. The smaller the KL divergence, the higher the approximation degree. Note that $\ln\left(P\left(P\right)\right)$ is only related to the observed data ***P*** and not to the parameters to be estimated. Therefore, maximizing *L*(*q*(***Ω***)) is equivalent to minimizing the KL divergence, so the variational distribution *q*(***Ω***) is an approximation of the posterior distribution $P\left(\Omega |P\right)$.

Based on the mean-field theory, the joint variational distribution composed of the parameters to be estimated is

(19) $$q\left(\Omega \right)=q\left(W,\alpha ,\beta \right)=q\left(W\right)q\left(\alpha \right)q\left(\beta \right)$$

By minimizing the KL divergence and utilizing the properties of conjugate priors, the variational posterior distributions of each parameter naturally take the following forms.

The prior of ***W*** satisfies a Gaussian distribution, so its variational posterior *q*(***W***) still follows a Gaussian distribution as

(20) $$q\left(W\right)=N\left(W|\mu ,\Sigma \right)$$

with

(21) $$\mu =\langle \beta \rangle \Sigma C^{T}P$$

(22) $$\Sigma ={\left(\mathrm{diag}\left(\langle \alpha \rangle \right)+\langle \beta \rangle C^{T}C\right)}^{-1}$$

where ***μ*** and ***Σ*** denote the mean and the posterior covariance matrix of the variational Gaussian distribution, respectively, and $\langle \cdot \rangle$ represents the expectation taken with respect to the corresponding variational distribution.

Similarly, the variational posteriors for the hyperparameters ***α*** and *β* also follow Gamma distributions as

(23) $$q\left(\alpha \right)=\prod_{i=1}^{2N}\Gamma \left(\alpha_{i}|a_{i},b_{i}\right)$$

(24) $$q\left(\beta \right)=\Gamma \left(\beta |\tilde{c},\tilde{d}\right)$$

where *a_i_* and *b_i_* represent the shape and rate parameters of the variational posterior *q*(*α_i_*) with i=1,2,…,2N, $\tilde{c}$ and $\tilde{d}$ represent the shape and rate parameters of the variational posterior *q*(*β*), respectively.

Under the variational Bayesian inference framework, a coordinate ascent algorithm [33] is employed to optimize the variational distributions. The resulting iterative update formulas for the parameters of each distribution are as follows

(25) $$a_{i}=a+1/2$$

(26) $$b_{i}=b+\frac{1}{2}\left(\mu_{i}^{2}+\Sigma_{ii}\right)$$

(27) $$\tilde{c}=c+M$$

(28) $$\tilde{d}=d+\frac{||P-C\mu |{|}^{2}+\mathrm{tr}\left(\Sigma C^{T}C\right)}{2}$$

(29) $$\langle \alpha_{i}\rangle =\frac{a_{i}}{b_{i}},\;\langle \beta \rangle =\frac{\tilde{c}}{\tilde{d}}$$

where *μ_i_* denotes the *i*-th element of the ***μ*** and *Σ_ii_* represents the *i*-th element of the main diagonal of the matrix ***Σ***.

For the variational Bayesian learning method, convergence is controlled by monitoring the lower bound *L*(*q*(***Ω***)) [45]

(30) $$\begin{array}{ll} L\left(q\left(\Omega \right)\right)= & \langle \ln\left(P\left(P|W;\beta \right)\right)\rangle +\langle \ln\left(P\left(W|\alpha \right)\right)\rangle +\langle \ln\left(P\left(\alpha \right)\right)\rangle \\ & +\langle \ln\left(P\left(\beta \right)\right)\rangle -\langle \ln\left(q\left(W\right)\right)\rangle -\langle \ln\left(q\left(\alpha \right)\right)\rangle -\langle \ln\left(q\left(\beta \right)\right)\rangle \end{array}$$

The computation of each term in *L*(*q*(***Ω***)) is highly computationally expensive, thus it needs to be simplified as:

(31) $$L\left(q\left(\Omega \right)\right)=-\frac{1}{2}\ln\langle \beta \rangle \left[||P-C\mu |{|}^{2}+\mathrm{tr}\left(\Sigma C^{T}C\right)\right]-\frac{1}{2}\sum_{i=0}^{2N}\langle \alpha_{i}\rangle \left(\mu_{i}^{2}+\Sigma_{ii}\right)+\mathrm{const}$$

where “const” is a constant term independent of the variables ***W***, ***α***, and *β*.

By iteratively updating all variational parameters and monitoring the change in *L*(*q*(***Ω***)), the algorithm is considered converged when its increment falls below a preset threshold. After the above iterative loop is completed, ***μ*** from Equation (21) gives the sparse coefficient vector ***w*** for the equivalent source strength:

(32) $$w=\mu \left(1:N\right)+i\mu \left(N+1:2N\right)$$

Then sound field reconstruction can be realized.

Algorithm 1 summarizes the framework of the proposed VBCS.

**Algorithm 1** Framework for VBCS
Input: ***P***, ***C***.Initialize: $\mu^{\left(0\right)}=0$, $\Sigma^{\left(0\right)}=I$, $a=b=c=d=10^{-6}$, the tolerance value: $\delta =10^{-6}$, maximum iterations $t_{\max}=100$.**While: **$\left|\frac{L_{t}\left(q\left(\Omega \right)\right)-L_{t-1}\left(q\left(\Omega \right)\right)}{L_{t-1}\left(q\left(\Omega \right)\right)}\right|>\delta$ and $t>t_{\max}$, **do**Calculate $\mu^{\left(t\right)}$ and according $\Sigma^{\left(t\right)}$ to Equations (21) and (22);Update the hyperparameters ${a_{i}}^{\left(t\right)}$, ${b_{i}}^{\left(t\right)}$, ${\tilde{c}}^{\left(t\right)}$ and ${\tilde{d}}^{\left(t\right)}$ according to Equations (25)–(28);
**End while**
Output *w* according to Equation (32).Calculate the reconstructed pressures using *w*.


### 2.3. Computational Complexity Analysis of VBCS

The proposed VBCS framework attains substantial computational efficiency by leveraging the Woodbury matrix identity to transform the inversion problem into a tractable low-dimensional computation as $\Sigma =D^{-1}-D^{-1}C^{T}{\left({\langle \beta \rangle }^{-1}I+CD^{-1}C^{T}\right)}^{-1}CD^{-1}$ in Equation (22), where $D=\mathrm{diag}\left(\langle \alpha \rangle \right)$ is a diagonal matrix. The complexity of VBCS algorithm is dominated by the iterative update of variational parameters in Algorithm 1. The asymptotic complexity of each core step is analyzed as follows:

1. Computation of ***C***^T^***P*** for updating the mean ***μ*** in Equation (21): Given $C\in R^{2M\times 2N}$ and $P\in R^{2M\times 1}$, the matrix-vector multiplication complexity is *O*((2*N*) × (2*M*)) = *O*(*MN*).
2. Computation of ***CD***^−1^***C***^T^:
  - $D=\mathrm{diag}\left(\langle \alpha \rangle \right)\in R^{2N\times 2N}$, so the computation complexity of ***D***^−1^ is *O*(2*N*) = *O*(*N*).
  - The product ***CD***^−1^: Since $C\in R^{2M\times 2N}$ is multiplied by diagonal matrix ***D***^−1^, the operation simplifies to element-wise column multiplication, with computational complexity *O*((2*N*) × (2*M*)) = *O*(*MN*).
  - The product ***CD***^−1^***C***^T^: When $CD^{-1}\in R^{2M\times 2N}$ is multiplied by $C^{T}\in R^{2N\times 2M}$, the computational complexity is *O*((2*M*) × (2*N*) × (2*M*)) = *O*(*M*^2^*N*).
3. Inversion of 2*M* × 2*M* matrix: The matrix $\left({\langle \beta \rangle }^{-1}I+CD^{-1}C^{T}\right)$ has dimensions 2*M* × 2*M*, and its inversion complexity is *O*((2*M*)^3^) = *O*(*M*^3^).
4. Update of hyperparameters ***α*** and *β* in Equations (25)–(28): The update relies on the diagonal elements ***μ*** and ***Σ***, involving 2*N* elements. The corresponding computational complexity is *O*(*N*).
5. Evidence lower bound (ELBO) calculation: Simplified to diagonal elements and vector norm computations, with complexity *O*(*N*).

Since *M* ≪ *N* in compressive sensing applications, the dominant computational complexity of the VBCS algorithm per iteration is *O*(*M*^2^*N + M*^3^) = *O*(*M*^2^*N*). This complexity results from the effective integration of the mean-field variational approximation and the Woodbury matrix identity. In contrast, the conventional BCS algorithm requires inversion of a 2*N* × 2*N* full covariance matrix, leading to an asymptotic complexity of *O*((2*N*)^3^) = *O*(*N*^3^).

Compared with conventional BCS, the VBCS algorithm achieves faster convergence speed and requires fewer iterations. This enhancement stems from fundamental differences in their posterior inference frameworks, computational complexity, and hyperparameter optimization strategies. Specifically, conventional BCS adopts a nested iterative strategy for the estimation of sparse signals and hyperparameters. It first fixes the hyperparameters to estimate the sparse signals via maximum likelihood estimation, and then updates the hyperparameters based on the estimated signals. This alternating iterative process repeats until both hyperparameters stabilize. However, high-dimensional signal estimation in each iteration involves complex matrix operations, resulting in substantial computational overhead, which ultimately leads to a large number of total iterations and slow convergence. In contrast, VBCS constructs a tractable approximate posterior distribution within a variational inference framework, transforming the intractable posterior inference into a deterministic optimization task that minimizes the Kullback–Leibler (KL) divergence between the approximate posterior and the true posterior. Utilizing the mean-field variational approximation, VBCS decouples the high-dimensional joint posterior of the signals and hyperparameters into a product of low-dimensional, independent marginal posteriors. It then applies the coordinate ascent algorithm to obtain closed-form solutions for single-variable subproblems, reducing the per-iteration computational complexity from *O*(*N*^3^) to *O*(*M*^2^*N*). Furthermore, VBCS incorporates hyperparameters into the variational posterior distribution for joint optimization with sparse signals, avoiding the nested iterative overhead inherent in BCS. Additionally, VBCS achieves efficient convergence determination by monitoring the change in the evidence lower bound (ELBO) and terminating iterations once the increase in ELBO falls below a preset threshold, thereby reducing the total convergence time.

Thus, by accelerating convergence and reducing the computational complexity from *O*(*N*^3^) to *O*(*M*^2^*N*), the VBCS algorithm markedly improves efficiency while preserving the advantages of Bayesian inference, offering a practical and viable solution for large-scale sound-field reconstruction.

## 3. Numerical Simulations

**Figure 2:**
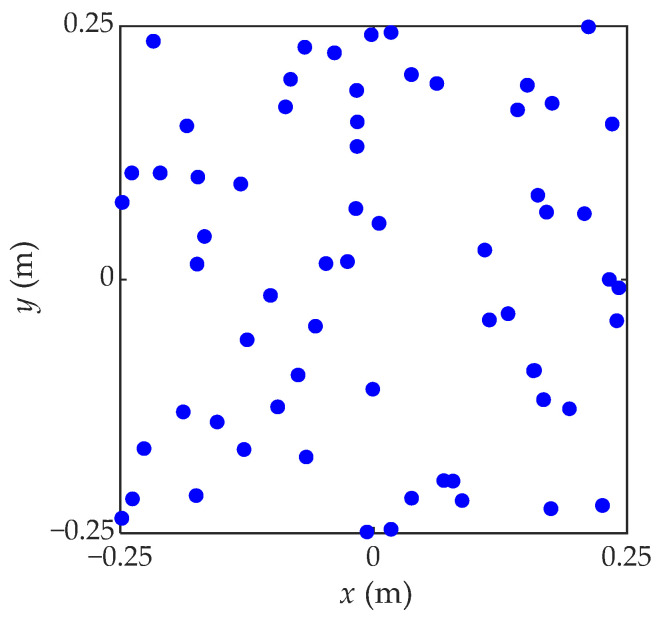
Simulated microphone array.

To validate the performance of the proposed VBCS algorithm in sound field reconstruction, numerical simulations were conducted. The simulated sound source comprised a simply supported steel plate with a thickness of 3 mm and dimensions of 0.5 × 0.5 m^2^. A harmonic force of 1 N amplitude excited the plate at its center, which was defined as the coordinate origin. The material properties of the steel plate were defined as follows: Poisson’s ratio of 0.28, density of 7.85 × 10^3^ kg/m^3^, and Young’s modulus of 2.1 × 10^11^ Pa. The theoretical solution of the sound field radiated from the vibrating steel plate is calculated using the Rayleigh’s integral. A measurement plane with the same in-plane size as the plate was located 0.05 m above the vibrating plate, where the spatial sampling interval was set to 0.025 m. A square-shaped random planar microphone array consisting of 64 microphones, as illustrated in Figure 2, was deployed on the measurement plane to capture the simulated sound pressure signals. To simulate real-world environmental interference, the collected sound pressures on the measurement plane were added with additive Gaussian white noise at an SNR of 30 dB. The reconstruction plane, which matched the measurement plane in in-plane dimensions, was placed 0.02 m away from the vibrating steel plate. Meanwhile, the equivalent source plane was located 0.05 m behind the vibrating plate, configured as a 21 × 21 grid with a spacing of 0.025 m, and its in-plane dimensions were consistent with those of the reconstruction plane.

To quantify the reconstruction accuracy of the sound pressure, the relative reconstruction error is defined as:

(33) $$\varepsilon_{\mathrm{rel}}=\frac{||P_{r}-P_{\mathrm{th}}|{|}_{2}}{||P_{\mathrm{th}}|{|}_{2}}\times 100\%$$

where ***P***_r_ and ***P***_th_ denote the reconstructed and theoretical sound pressure vectors, respectively.

The influence of hyperparameter settings (*a*, *b*, *c*, *d*) in the Gamma priors of the Bayesian hierarchical model within the proposed VBCS algorithm on sound field reconstruction performance is first investigated. To adhere to the core principle of non-informativeness, these hyperparameters are conventionally set to very small positive values, constituting weakly informative priors. As these hyperparameters approach zero, the Gamma distribution converges asymptotically to a log-uniform distribution over its domain. This configuration imposes almost no preference for any positive scale of parameter values, thereby ensuring that subsequent statistical inferences remain driven by observed data. Crucially, setting these values sufficiently small effectively approximates a non-informative state while keeping them strictly positive, which satisfies the positivity requirement of the distribution and helps to avoid potential numerical instability issues arising from boundary singularities.

To examine the sensitivity of the Gamma prior hyperparameters (*a*, *b*, *c*, *d*) in the proposed VBCS algorithm under high-noise conditions, simulations were performed at an SNR of 10 dB with a series of initial values ranging from 10^−1^ to 10^−8^.

**Figure 3:**
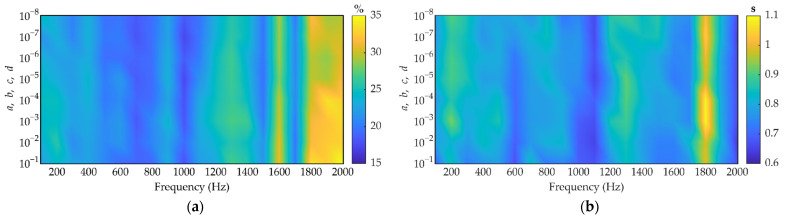
Reconstruction error and runtime of VBCS versus initial hyperparameters and frequency with an SNR of 10 dB: (**a**) reconstruction error; (**b**) runtime.

Figure 3a illustrates the variation in reconstruction error versus hyperparameters (*a*, *b*, *c*, *d*) across the frequency range of 200–2000 Hz. The most prominent feature is the near-vertical contour distribution of reconstruction errors, indicating that at any given frequency, the color-coded error values change very gradually along the vertical axis (i.e., with the values of hyperparameters). Significant changes in error occur primarily along the horizontal axis (i.e., with increasing frequency, especially above 1000 Hz). Conversely, along the vertical axis, the error values remain relatively uniform across multiple orders of magnitude for most frequencies. This demonstrates that the reconstruction accuracy of the proposed VBCS algorithm is extremely insensitive to the specific settings of this set of Gamma prior hyperparameters.

Figure 3b depicts the variation in runtime of the proposed algorithm with hyperparameters over the frequency range of 200–2000 Hz. It can be observed that for most hyperparameter settings, the runtime increases with frequency, approaching 1.1 s in high-frequency regions while remaining relatively stable at approximately 0.6 s at the low-to-mid frequencies. This frequency-dependent computational overhead arises from increased coherence in the sensing matrix at high frequencies, necessitating more variational inference iterations to meet the convergence threshold. Along the hyperparameter axis, the runtime varies continuously and gradually, devoid of abrupt discontinuities near specific values. This confirms high robustness and consistency in computational efficiency within the hyperparameter variation range.

Given the low sensitivity of VBCS to initial hyperparameters in both reconstruction accuracy and computational efficiency, as well as its overall stable performance, investing substantial computational resources in fine-tuning these hyperparameters yields limited benefits. Therefore, for all subsequent simulations and experiments in this study, the Gamma prior hyperparameters (*a*, *b*, *c*, *d*) of the VBCS algorithm are uniformly initialized to 10^−6^.

**Figure 4:**
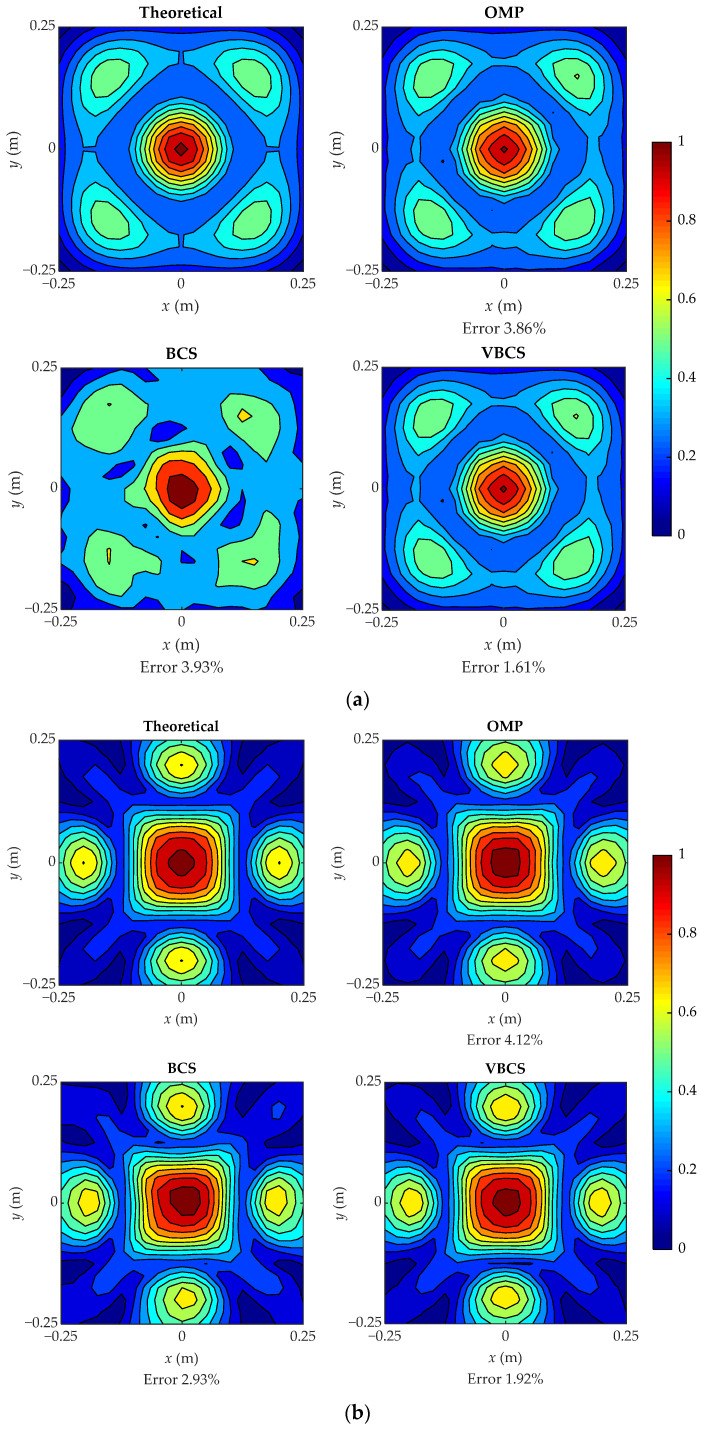

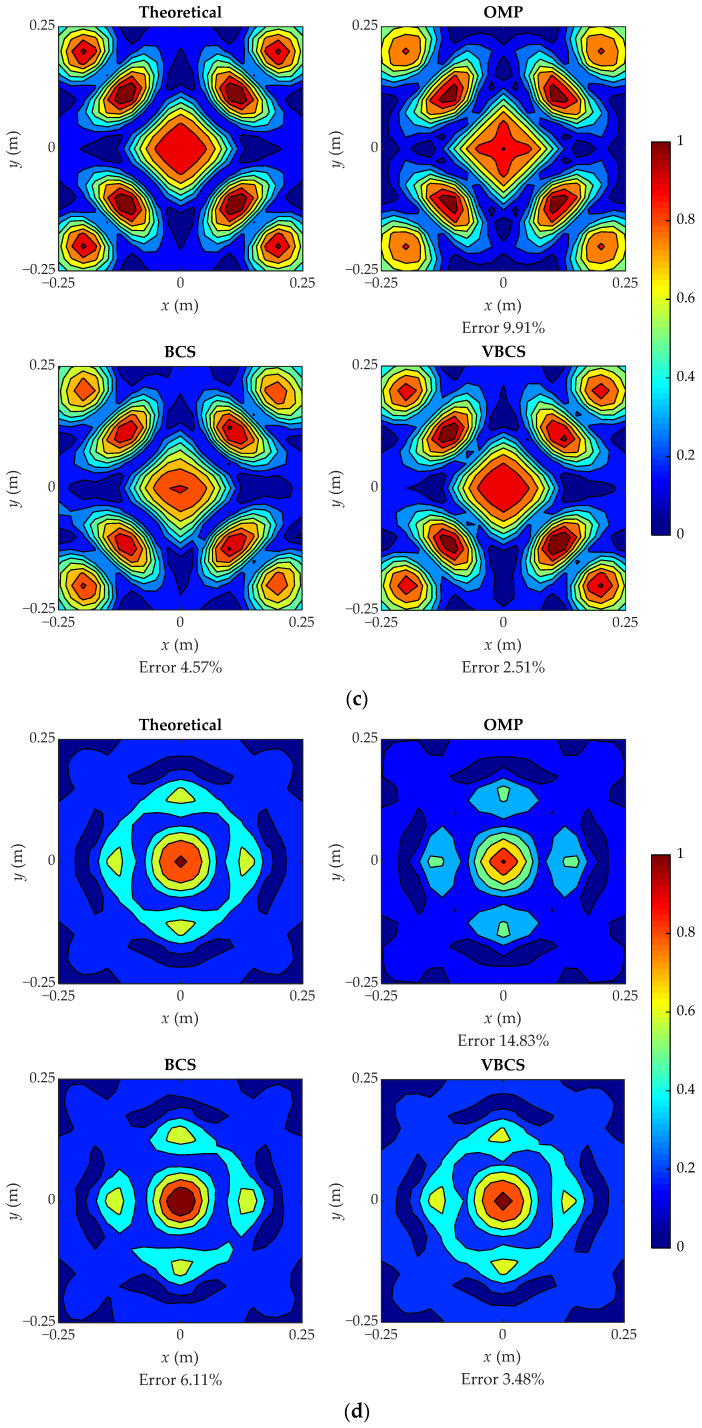

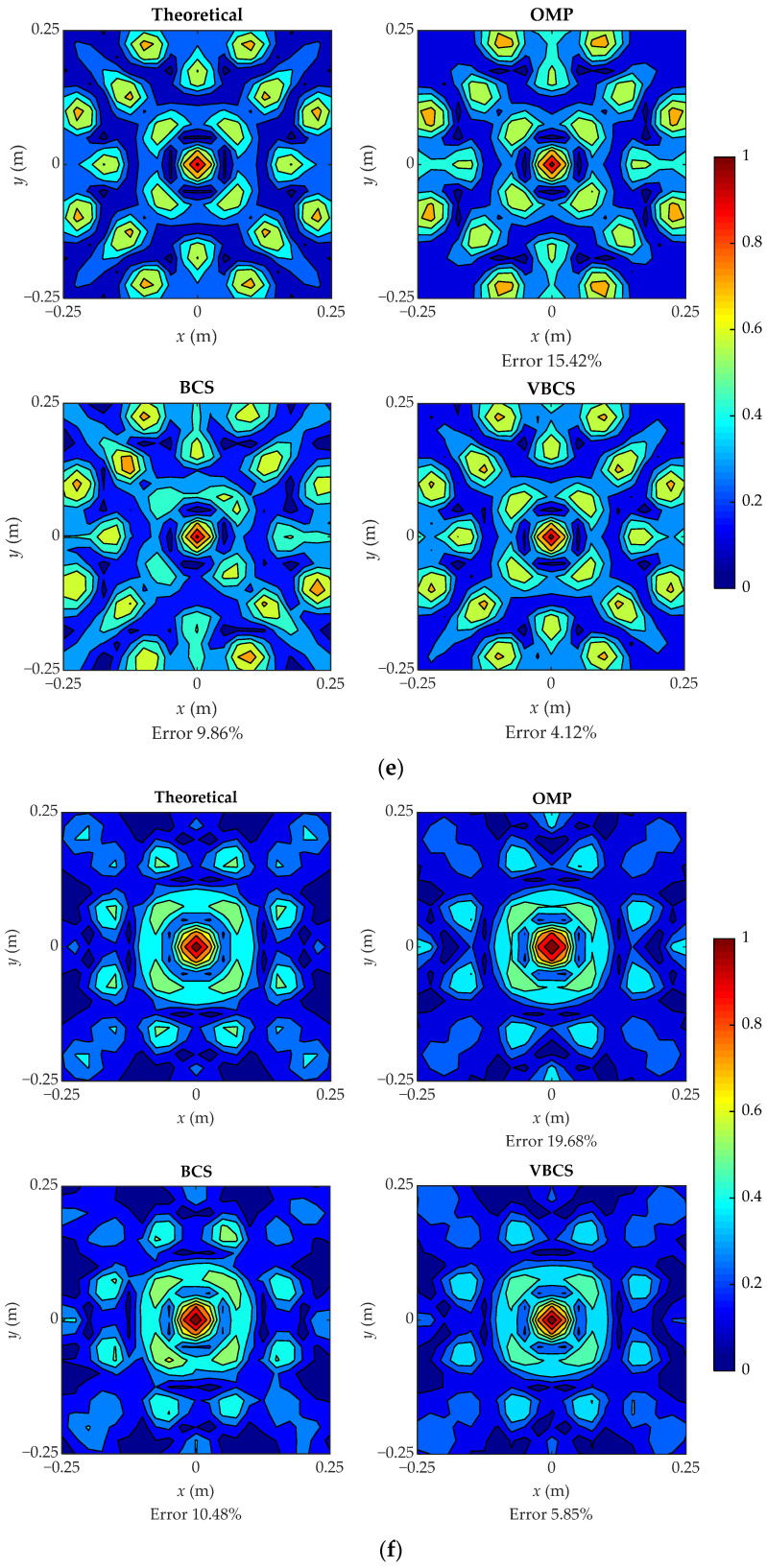

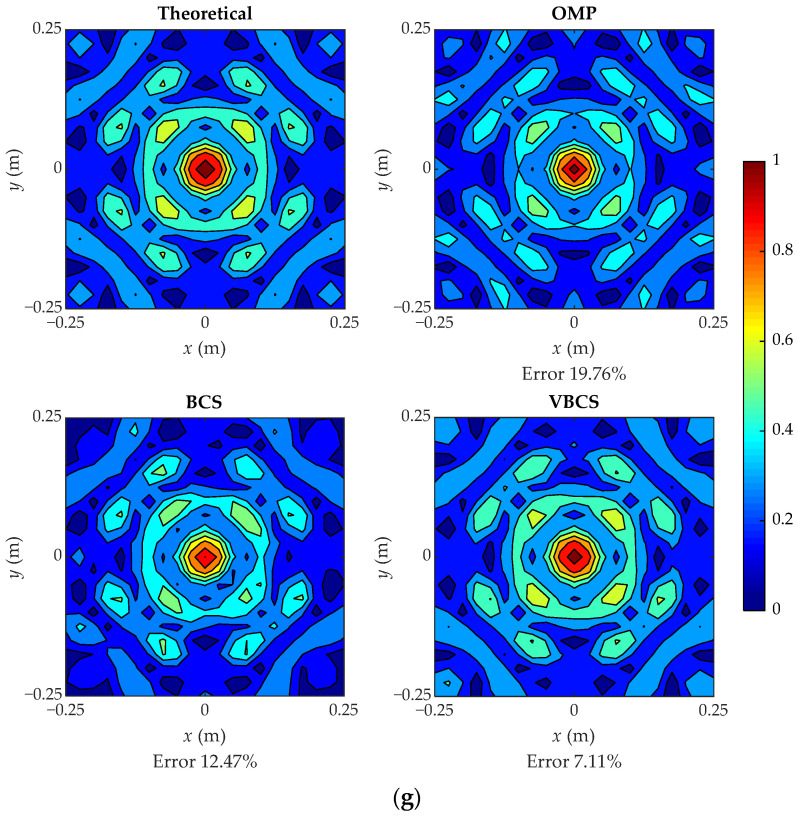
Comparison of reconstructed sound pressures with 64 sampling points at seven frequencies: (**a**) 300 Hz; (**b**) 600 Hz; (**c**) 900 Hz; (**d**) 1200 Hz; (**e**) 1500 Hz; (**f**) 1800 Hz; (**g**) 2000 Hz.

To evaluate the performance advantages of the proposed VBCS algorithm in broadband sound pressure reconstruction, the sound pressure reconstruction results of VBCS are compared with those of BCS and OMP across the frequency range of 100 Hz to 2000 Hz. Figure 4 presents a comparison of the three algorithms in sound pressure reconstruction using 64 random sampling points, at frequencies of 300 Hz, 600 Hz, 900 Hz, 1200 Hz, 1500 Hz, 1800 Hz, and 2000 Hz. To facilitate observation of the spatial distribution of sound pressure, normalized sound pressure was adopted, ensuring that the displayed amplitudes are confined to the range [0, 1]. The results indicate that all three methods achieved reconstruction with relatively low errors across different frequencies. As depicted in Figure 4a,b, the reconstruction errors of the three methods are extremely small at 300 Hz and 600 Hz. Specifically, the errors of the OMP method are 3.86% and 4.12%, respectively, while those of the BCS method are 3.93% and 2.93%. In contrast, the VBCS method exhibits smaller errors, only 1.61% and 1.92%, indicating its superior reconstruction performance in the low-frequency range. From Figure 4c–e, it can be seen that at 900 Hz, 1200 Hz, and 1500 Hz, the BCS method cannot accurately reconstruct the sound pressure field, as it fails to restore the peak values and symmetric distribution characteristics. The reconstruction errors for the OMP method are 9.91%, 14.83%, and 15.42%, respectively, whereas the errors for the BCS method are 4.57%, 6.11%, and 9.86%. By comparison, the VBCS method exhibits progressively better performance in the mid-frequency range, with errors as low as 2.51%, 3.48%, and 4.12%. Moreover, VBCS accurately recovers the peak values and symmetric distribution, demonstrating its superiority over both the BCS and OMP methods. As shown in Figure 4f,g, at high frequencies of 1800 Hz and 2000 Hz, OMP yields high errors of 19.68% and 19.76%, and BCS yields 10.48% and 12.47%, whereas VBCS reduces these to 5.85% and 7.11%. The results of OMP and BCS suffer from significant degradation, losing edge details and symmetry, while VBCS preserves the spatial distribution of the sound field and accurately reconstructs key features. Regarding reconstruction error, the error growth of both the OMP and BCS methods is significantly steeper than that of the VBCS method. Notably, the VBCS error itself remains stable at below 10% throughout the high-frequency range. In summary, although effective reconstruction is possible with all three methods, the proposed VBCS method demonstrates superior overall accuracy in sound field reconstruction.

**Figure 5:**
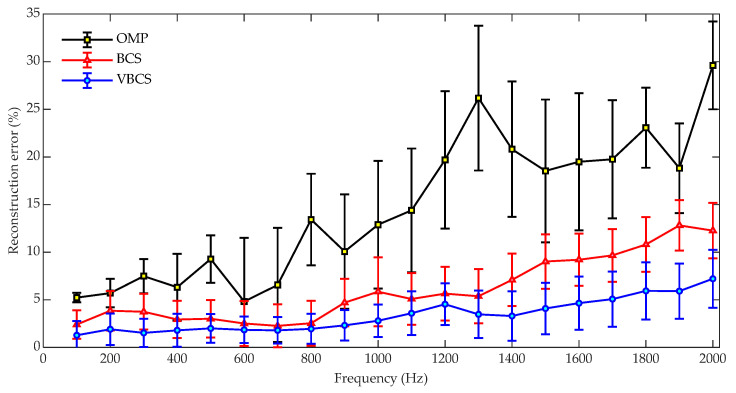
Comparison of reconstruction errors versus frequency for the three methods.

Figure 5 shows a comparison of the sound field reconstruction errors for the OMP, BCS, and VBCS algorithms across the 100–2000 Hz frequency range. In the simulations, the sound field was reconstructed using the sound pressure from 64 randomly selected measurement points out of a total of 441 points on the hologram surface. Independent Gaussian white noise with an SNR of 30 dB was added to the measured pressures. For a statistical evaluation, 100 independent simulation trials were conducted for each algorithm, with parameters initialized according to their respective default strategies. Noted that in any given trial, all algorithms used the identical random microphone array configuration and noise sample to ensure a fair comparison. All simulation experiments were performed on a computer equipped with an Intel i5-1135G7 CPU and 16 GB of RAM. Finally, the performance of each algorithm is presented as the statistical mean of the results from the 100 independent trials, with error bars indicating the standard deviation. To ensure the strict fairness of the performance comparison among the three algorithms, this study adopts the following core principles: in each independent trial, all algorithms involved in the comparison must use identical sampling masks (i.e., sampling point positions), measurement data, and initialization parameters. This ensures that the observed performance differences are attributed solely to the inherent characteristics of the algorithms themselves, rather than random conditions or discrepancies in initialization. For the numerical simulation, two factors are randomized to ensure scenario coverage: the sampling mask is independently generated in each trial by randomly selecting a preset number of sampling points from all measurement points on the hologram plane, and additive white Gaussian noise based on independent random seeds is introduced to simulate the randomness of real-world noise. Meanwhile, fixed factors are strictly controlled: a unified set of initialization parameters is adopted across all trials, and the operational procedure is kept consistent. For a single trial, the randomly generated sampling point positions and noise realizations are first fixed to generate measurement data; subsequently, the data corresponding to this mask is fed into the three algorithms for reconstruction. This approach avoids performance deviations caused by differences in sampling distribution or noise. As shown in Figure 5, the reconstruction error of all methods exhibits an overall upward trend with increasing frequency. OMP remains near 5% at low frequencies, then surges with large fluctuations to almost 30% at 2000 Hz. BCS demonstrates intermediate error performance, with its error not exceeding 13% across the entire frequency band. In contrast, VBCS achieves the lowest reconstruction error, stably maintaining values below 8% across all frequencies and exhibiting the most stable performance. In terms of the fluctuation in reconstruction performance, as indicated by the error bars, OMP exhibits the largest variations, particularly within the 1200 Hz to 1800 Hz range. Conversely, VBCS shows the smallest fluctuations, highlighting its robustness to frequency changes. These results demonstrate that VBCS outperforms both OMP and BCS in terms of reconstruction accuracy and stability, with particularly outstanding performance at high frequencies.

**Figure 6:**
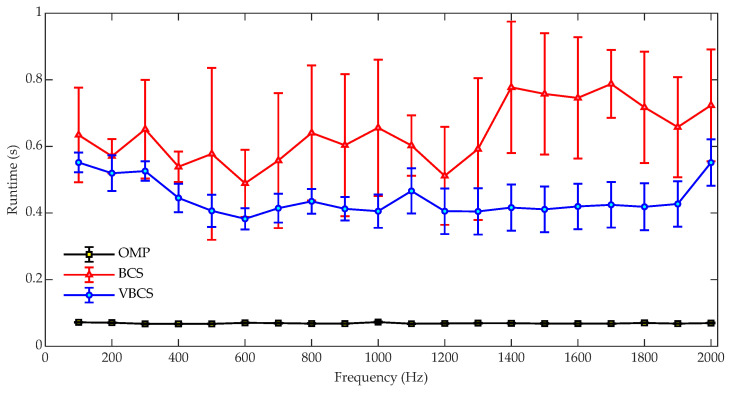
Comparison of computational runtimes versus frequency for the three methods.

Figure 6 compares the runtimes of OMP, BCS, and VBCS for sound field reconstruction over the 100–2000 Hz band. The OMP algorithm maintains the lowest and most stable runtime, remaining consistently around 0.07 s with minimal fluctuations, indicating its high computational efficiency and insensitivity to frequency variations. In contrast, the BCS algorithm exhibits significantly higher and more variable computational costs. Its runtime shows considerable fluctuations with frequency, accompanied by relatively large error bars, which reflects its instability and high sensitivity to specific frequency conditions. The proposed VBCS algorithm strikes a balance between the two, demonstrating a moderate runtime that generally ranges from approximately 0.3 to 0.5 s across most frequencies. While typically higher than that of the OMP algorithm, it is substantially lower and more stable than that of the BCS method. The relatively small error bars for VBCS further indicate consistent and predictable computational performance. Overall, while the OMP algorithm achieves the highest computational efficiency, its reconstruction accuracy is lower compared with those of the other two methods. The VBCS algorithm, in contrast, achieves a favorable trade-off between reconstruction accuracy and computational efficiency, addressing the accuracy limitations of OMP while mitigating the computational burden associated with the BCS method.

**Figure 7:**
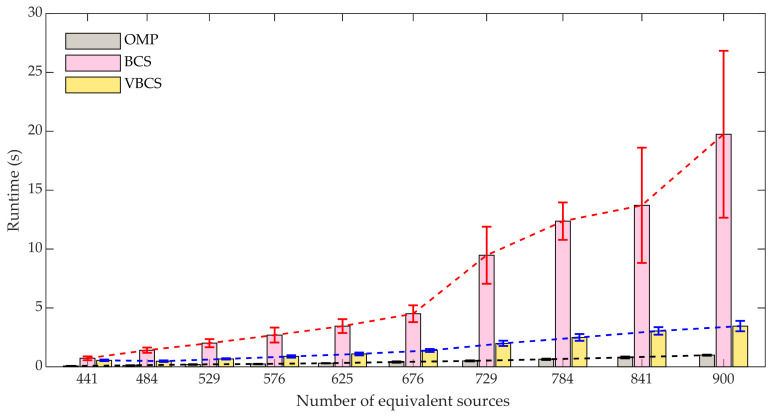
Comparison of runtimes of the three methods versus the number of equivalent sources.

To further evaluate computational efficiency, the runtime variation with the number of equivalent sources *N* was investigated at an SNR of 30 dB and a frequency of 2000 Hz. To assess the robustness of the three methods, 100 independent trials were conducted, each utilizing 64 sampling points. Figure 7 depicts the runtimes of the OMP, BCS, and VBCS algorithms as the equivalent source number *N* increases from 441 to 900. It can be observed that the runtime of all three algorithms exhibits an upward trend with the increase in the equivalent source number. The OMP algorithm maintains an extremely low runtime with no significant fluctuations as *N* increases. This is attributed to its concise algorithm structure based on greedy iteration, which performs sparse reconstruction solely through local atom selection, resulting in lower computational complexity. Consequently, OMP demonstrates more stable temporal performance even when *N* is large. With an increase in *N*, the runtime of the BCS algorithm rises drastically with substantial fluctuations, and the corresponding error range expands progressively. This indicates that the algorithm suffers from reduced computational efficiency and degraded stability when the equivalent source number is high. The VBCS algorithm exhibits a relatively moderate increase in runtime, which demonstrates that the incorporation of variational inference optimizes the probability distribution update process throughout the iterative procedure. By avoiding the complex matrix operations inherent in BCS, VBCS realizes a significant improvement in computational efficiency. These results demonstrate that the time complexity scaling of each algorithm with the number of equivalent sources *N* is dictated by its underlying computational strategy. Specifically, the global optimization framework of BCS prioritizes reconstruction accuracy, incurring a substantial computational cost that grows superlinearly with *N*. In contrast, VBCS employs variational inference to strike a balance between precision and efficiency, resulting in a more favorable scaling. The OMP algorithm, by relying on greedy local optimization, achieves the lowest time complexity at the expense of some reconstruction accuracy.

**Figure 8:**
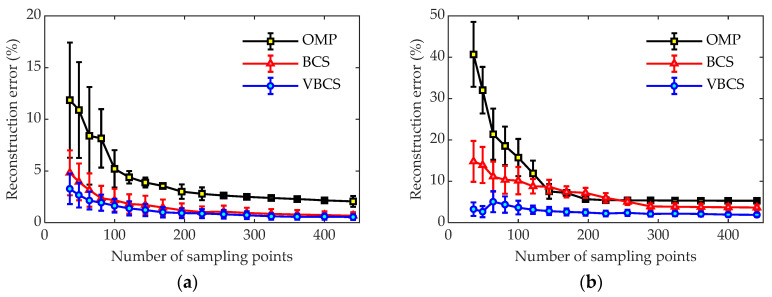
Reconstruction errors of the three methods under different numbers of sampling points: (**a**) 700 Hz and (**b**) 1700 Hz.

To investigate the relationship between the number of sampling points and sound field reconstruction accuracy, the reconstruction performance with 16 different sampling point configurations on the measurement plane was systematically examined. Figure 8 compares reconstruction errors versus the number of sampling points at 700 Hz and 1700 Hz under 30 dB SNR. It can be seen that all three algorithms exhibited decreasing reconstruction errors with increasing sampling points. As shown in Figure 8a, the reconstruction error of the VBCS algorithm remains consistently lower than that of the OMP and BCS algorithms as the number of sampling points gradually increases. At 36 sampling points, the reconstruction error of VBCS is only 3.2%, in contrast to 4.8% for BCS and 11.9% for OMP. As the number of sampling points continues to increase, the reconstruction error of OMP remains at 2.06%, whereas the errors of both VBCS and BCS eventually stabilize at approximately 0.5%. Figure 8b shows that the VBCS algorithm consistently exhibits superior stability and accuracy to the OMP and BCS algorithms. At 1700 Hz, with merely 36 sampling points, VBCS yields a reconstruction error of only 3.3%, compared to 14.8% for BCS and 40.7% for OMP. When the number of sampling points is increased to 441, the reconstruction error of OMP remains the highest at 5.3%, while BCS and VBCS achieve lower errors of 3.7% and 1.9%, respectively. This comparison indicates that VBCS outperforms OMP and BCS in sound field reconstruction at both low and high frequencies, especially under sparse sampling conditions. This makes it a promising candidate for wideband acoustic applications that demand high accuracy with minimal sampling points.

**Figure 9:**
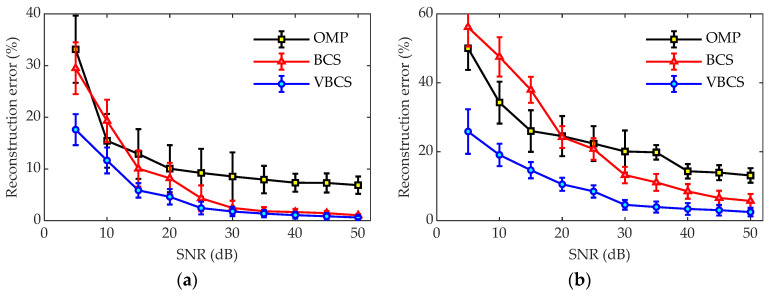
Reconstruction errors of the three methods under different SNRs: (**a**) 700 Hz and (**b**) 1700 Hz.

To evaluate the noise robustness of the three algorithms under different SNR levels, simulations were performed at 700 Hz and 1700 Hz with 64 measurement points, as shown in Figure 9. As the SNR increases from 5 dB to 50 dB, the reconstruction error of all algorithms gradually decrease, with VBCS consistently demonstrating lower errors than OMP and BCS. Specifically, OMP yields the highest reconstruction error across nearly the entire SNR range, indicating its limited robustness, especially under low-SNR condition. BCS shows moderate performance, with consistently lower errors than OMP but still higher than those of the VBCS. In contrast, the proposed VBCS method achieves the lowest reconstruction error at all tested SNRs, highlighting its superior accuracy and noise resilience. Error bars at each SNR point show that VBCS exhibits smaller error fluctuations, implying greater stability compared to OMP and BCS. Overall, the results confirm the effectiveness of the variational Bayesian framework adopted in VBCS for robust sound field reconstruction under noisy environments.

## 4. Experiments

**Figure 10:**
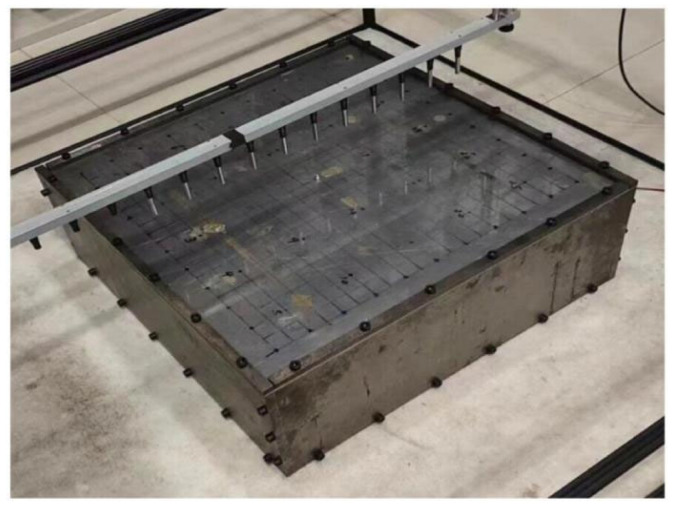
Experimental setup.

**Figure 11:**
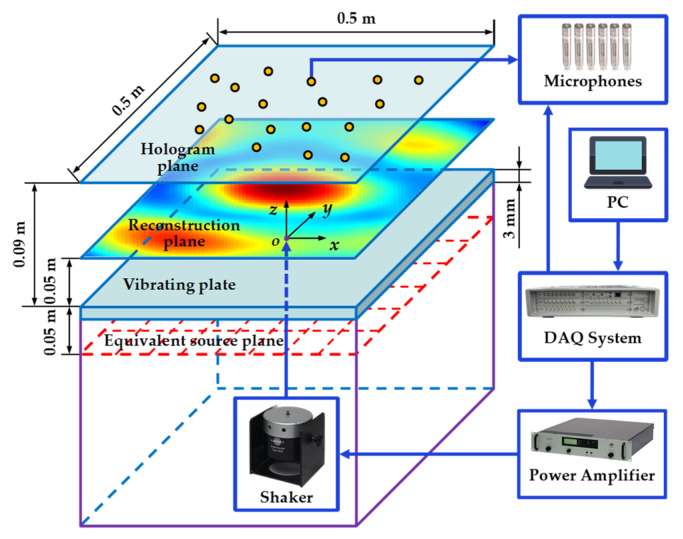
Schematic of the experimental spatial configuration.

Experiments were conducted within a semi-anechoic chamber to further validate the performance of the proposed VBCS method. The experimental setup is depicted in Figure 10. A steel plate with dimensions of 0.5 × 0.5 m^2^ and a thickness of 3 mm served as the radiating sound source. The plate was excited at its center by an electrodynamic shaker driven by a power amplifier from Brüel and Kjær. The excitation was a multi-tone signal, synthesized by a series of harmonic signals, with a frequency range of 100–2000 Hz and a frequency interval of 100 Hz. This signal was used to drive the shaker, ensuring the plate was simultaneously excited by all frequency components. This configuration simulated broadband acoustic excitation in practice, thereby validating the effectiveness of the proposed method for processing multi-frequency acoustic field data. The experiment was conducted using the LMS Test. Lab test system from Siemens and partial equipment from Brüel and Kjær. The hardware equipment utilized is listed in Table 1. Sound field scanning was performed on two parallel planes positioned above the plate (each 0.5 m × 0.5 m) using a linear array of 11 46AE microphones (GRAS Sound and Vibration). As shown in Figure 11, the hologram plane and the reconstruction plane were positioned 0.09 m and 0.05 m above the plate surface, respectively. Each measurement plane contained 121 points arranged in a uniform 11 × 11 grid with a spacing of 0.05 m. Both the excitation signal and the microphone sound pressure signals were synchronously acquired at a sampling frequency of 10.24 kHz for a duration of 5 s. To ensure phase consistency in sound pressure measurements and eliminate phase mismatch between the excitation signal and the measured sound pressure signals, the excitation signal served as a phase reference during scanning. Despite employing simultaneous multi-tone excitation, a sequential single-frequency reconstruction strategy was implemented for sound field. Specifically, after synchronously acquiring time-domain sound pressure signals from the microphone array, a fast Fourier transform (FFT) decomposed both the reference excitation signal and the measured sound pressure signals into discrete frequency components. For each frequency, the reference signal phase was subtracted from the sound pressure phase at each measurement point. This process ensures that the corrected sound pressure phase accurately reflects the intrinsic phase characteristics of the sound field while maintaining full synchronization with the excitation signal, thereby fundamentally eliminating phase mismatch in single-frequency sound field reconstruction. A planar array of 121 equivalent sources, uniformly distributed on a plane parallel to and 0.05 m behind the steel plate, also in an 11 × 11 grid with 0.05 m spacing, was employed. From the 121 sampling points within the hologram plane, a subset of 64 points was randomly selected as the input for each reconstruction method to recover the sound pressure at all 121 points on the reconstruction plane. The directly measured sound pressures at these 121 reconstruction plane points constituted the reference data for evaluating the performance of the three methods.

**Figure 12:**
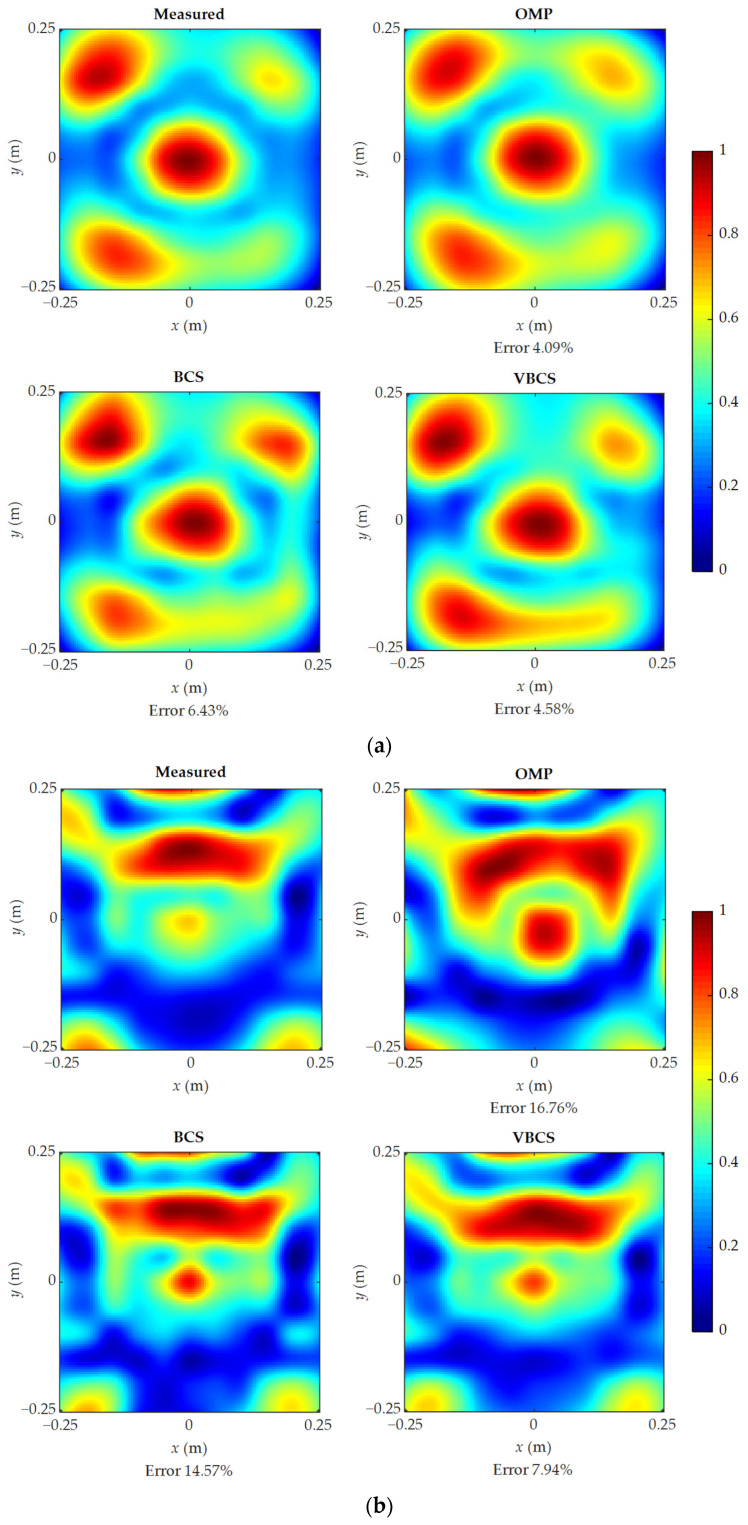

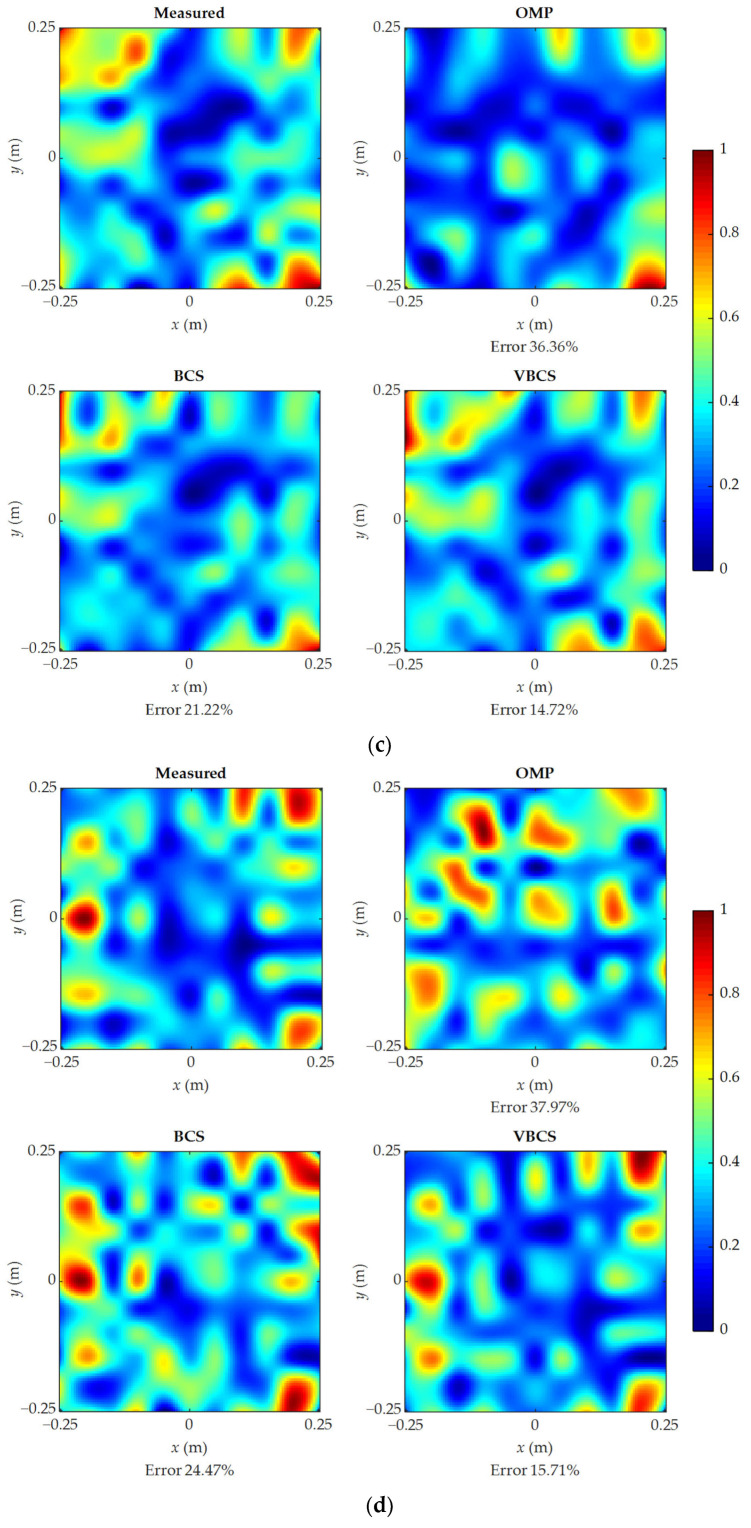
Comparison of reconstructed sound pressures with 64 sampling points at four frequencies: (**a**) 300 Hz; (**b**) 900 Hz; (**c**) 1600 Hz; (**d**) 2000 Hz.

Figure 12 compares the reconstructed and measured sound pressures for the three methods at 300 Hz, 900 Hz, 1600 Hz, and 2000 Hz. To eliminate interference from absolute pressure magnitudes and focus on evaluating the accuracy of reconstruction algorithms in recovering spatial sound field patterns, normalized sound pressure is employed to compare reconstructed and measured distributions. The results show that all methods achieve good agreement with the measured sound pressure distributions at 300 Hz and 900 Hz. Notably, the VBCS method yields smaller reconstruction errors of 4.58% and 7.94% at 300 Hz and 900 Hz, respectively, compared to 4.09% and 16.76% for OMP, and 6.43% and 14.57% for BCS. At 1600 Hz, the VBCS method achieves a reconstruction error of 14.72%, significantly lower than that of OMP at 36.36% and that of BCS at 21.22%. At 2000 Hz, where the sound field becomes highly complex, OMP and BCS exhibit reconstruction errors of 37.97% and 24.47%, respectively, whereas VBCS maintains superior performance with an error of only 15.71%. The above results demonstrate that the proposed VBCS method delivers stable and superior reconstruction performance across a broad frequency range, offering an effective solution for practical high-accuracy sound field reconstruction.

**Figure 13:**
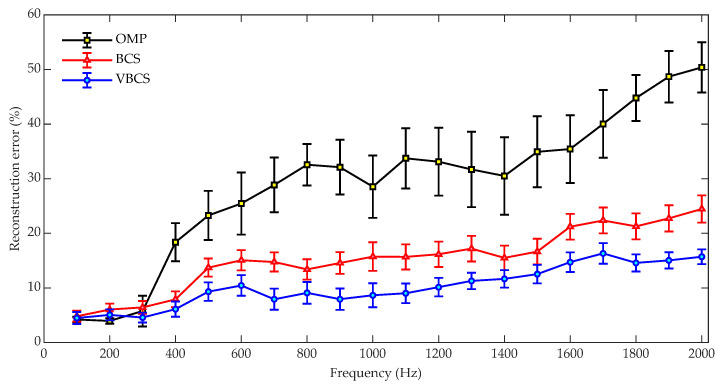
Reconstruction errors versus frequency for the three methods.

To conduct a statistical evaluation, 1000 independent trials were performed for each of the three algorithms to compare their sound field reconstruction error and runtime. In each trial, 64 measurement points were randomly selected from the 121 sensors on the hologram plane for sound pressure acquisition and subsequent sound field reconstruction. All three algorithms were operated on the identical measurement matrix to ensure a fair comparison. The reconstruction performance was presented as the average reconstruction error and average runtime over the 1000 trials, with error bars denoting the corresponding standard deviations. In the experimental verification, the randomized factor is limited to the sampling mask, which is created by randomly selecting 64 points out of 121 measurement points on the hologram plane. The fixed factors are as follows: (i) only the inherent background noise of the experimental environment is used (without adding artificial noise); (ii) a unified set of initialization parameters is applied throughout. During the operational procedure, the randomly generated sampling mask is fixed for each trial; the sensors at the positions specified by this mask are then used to collect data, which is subsequently input into the algorithms for reconstruction. This effectively eliminates the interference of sampling point distribution differences on the experimental results. Figure 13 compares the sound field reconstruction errors of OMP, BCS and VBCS across the frequency range of 100–2000 Hz. The OMP method exhibits the poorest robustness, with its reconstruction error increasing markedly with frequency and exceeding 50% at 2000 Hz. The large error bars further indicate significant fluctuations in reconstruction accuracy, reflecting limited capability to handle rising acoustic complexity at higher frequencies. BCS demonstrates moderate performance, consistently surpassing OMP but remaining less accurate than VBCS. Its error grows steadily from 5% at low frequencies to 24% at 2000 Hz, with narrower error bars than OMP. In contrast, the VBCS method achieve the lowest error and highest stability across the entire frequency range. In summary, VBCS significantly surpasses both OMP and BCS in accuracy and robustness, demonstrating clear advantages for broadband sound field reconstruction, especially under demanding high-frequency conditions.

Furthermore, as depicted in Figure 13, the reconstruction error of the experimental data exhibits an upward trend as frequency increases. This phenomenon can be primarily attributed to the following three reasons:

1. At high frequencies, the measured acoustic pressure signals are highly susceptible to a particularly severe loss of the information essential for sound field reconstruction. The evanescent waves, which carry high-resolution spatial details of high-frequency sound fields, decay exponentially with propagation distance. Often, before reaching the measurement array, their amplitude falls below the background noise level, meaning that the high-frequency information necessary for reconstruction is effectively lost at the acquisition stage. Moreover, according to the spatial sampling theorem, capturing high-frequency components requires the microphone spacing to be smaller than half the acoustic wavelength. Given the very short wavelengths at high frequencies, practical arrays often violate this criterion, leading to spatial aliasing—where high-frequency components are folded into lower frequencies—and thus irreversible loss of information during sampling. Even in the ideal absence of attenuation and aliasing, the low energy of high-frequency signals makes them particularly vulnerable to being overwhelmed by measurement and environmental noise, further degrading the usable information in the recorded data. It must be emphasized that under hardware constraints, the performance degradation in high-frequency sound field reconstruction stems from the inherent information deficit of the inverse problem itself. This represents a universal limitation for all compressed sensing-based NAH methods, rather than a defect of any specific algorithm.
2. The wavelength of sound waves is reduced at high frequencies, leading to diminished discrepancies in propagation paths between equivalent sources and measurement points. This similarity in propagation characteristics causes the transfer functions to converge, which enhances the correlation among column vectors of the sensing matrix. At higher frequencies, reduced acoustic wavelengths diminish propagation path differences between equivalent sources and measurement points. This results in greater similarity among transfer functions, thereby enhancing correlation between column vectors of the sensing matrix. Both the cross-correlation coefficient and condition number of the sensing matrix increase with rising frequency, compromising the incoherence requirement that is fundamental to the validity of compressive sensing.
3. The core premise of the ESM within the CS framework is that the sound field can be sparsely represented by a small number of equivalent sources. This approximation yields high accuracy at low frequencies, where the difference in radiation characteristics between equivalent sources and the actual sound source is negligible. In contrast, high-frequency sound fields encompass more high-order acoustic modes and high spatial frequency components, rendering the sound field distribution more complex. As a result, the sparsity assumption of equivalent sources is consequently weakened, and the proportion of non-zero elements in the equivalent source strength vector increases with frequency. This ultimately degrades the representational accuracy of the basis functions.

**Figure 14:**
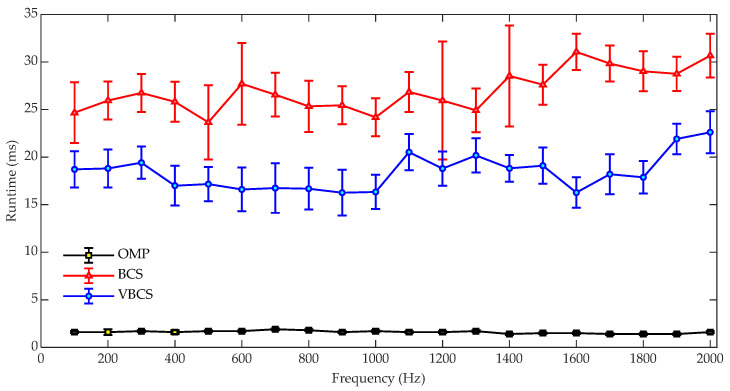
Computational runtimes versus frequency for the three methods.

Figure 14 shows the computational runtimes of the OMP, BCS, and VBCS algorithms over the 100–2000 Hz frequency range. The OMP algorithm demonstrates the lowest runtime, consistently remaining near 2 ms with negligible fluctuations as frequency increases. This high computational efficiency stems from its greedy selection mechanism employed by OMP, which significantly reduces iterative complexity compared to Bayesian approaches. Conversely, the BCS algorithm exhibits the highest runtime, ranging from 25 ms to 35 ms, resulting from the intensive computations required by its Bayesian inference framework. In contrast, VBCS achieves a moderate execution time, ranging between 15 ms and 20 ms. Although VBCS also adopts a Bayesian framework, it employs an optimized variational inference procedure that enables a more efficient approximation of posterior distributions, thereby reducing computational complexity compared to BCS and leading to faster convergence and lower runtime. Consequently, VBCS achieves a superior balance among reconstruction accuracy, computational efficiency, and robustness under varying conditions, making it more suitable for practical sound field reconstruction tasks requiring both high accuracy and real-time performance.

**Figure 15:**
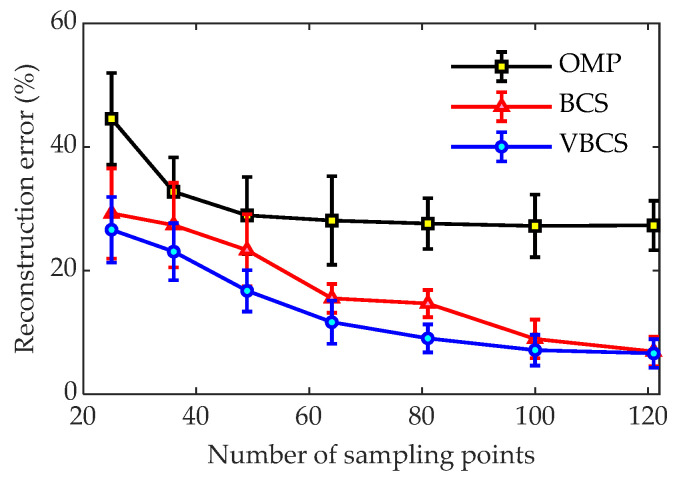
Reconstruction errors of the three methods under different numbers of sampling points at 1400 Hz.

Figure 15 compares the reconstruction errors of the three methods versus the number of sampling points, with the frequency fixed at 1400 Hz to isolate this variable. Under identical sampling conditions, VBCS consistently yields the lowest errors, demonstrating a clear accuracy advantage. Specifically, with 121 sampling points, both VBCS and BCS achieve relatively low reconstruction errors, stabilizing around 6%, while the error for the OMP algorithm remains above 27%. When the number is reduced to 64 points, the reconstruction error for OMP exceeds 28%, that for BCS decreases to about 15%, and VBCS drops to around 11%. With only 25 sampling points, the reconstruction error for OMP is as high as 42%, BCS is close to 30%, and VBCS remains the lowest at approximately 26%. Overall, VBCS delivers the superior reconstruction performance over OMP and BCS across all sampling densities. Notably, VBCS maintains excellent performance even with very few sampling points, which means the number of deployed sensors can be significantly reduced in engineering applications, thereby providing technical support for the development of cost-effective and efficient acoustic testing systems.

## 5. Discussion

Previous sections systematically validated the superior accuracy and computational efficiency of the VBCS algorithm compared to BCS and OMP in the sound field reconstruction. However, constrained by its model assumptions and design principles, VBCS still exhibits performance limitations.

The variational Bayesian inference framework employed in this study is based on the mean-field approximation, which posits mutual independence among the variational posterior distributions of the equivalent source sparse coefficients. This assumption decomposes the high-dimensional joint posterior into a product of lower-dimensional marginal distributions, significantly reducing computational complexity and making it suitable for sound field reconstruction with large-scale equivalent sources.

Nevertheless, when sound sources exhibit strong coherence, significant statistical correlations exist among the equivalent source coefficients. The independence assumption of the mean-field assumption weakens such correlations, leading to the variance of the variational posterior distribution being slightly smaller than that of the true posterior distribution. Theoretically, the magnitude of this underestimation effect is positively correlated with the coherence of sound sources. For single sources or weakly coherent multiple sources, coefficient correlations are weak, and the uncertainty underestimation is negligible. In contrast, for multi sources with strong coherence, neglecting the correlations causes the variance of the variational posterior distribution to deviate from the true posterior distribution, which may affect the interpretation of uncertainty quantification results.

This does not imply that VBCS is inherently inferior to BCS. Results presented in this study demonstrate that VBCS outperforms BCS in both reconstruction accuracy and computational efficiency. Although the mean-field assumption ignores coefficient correlations, introducing approximation error, in the scenarios where VBCS is applicable, this error is completely offset by the adaptive advantages of its variational framework, even yielding higher accuracy. The reasons are threefold: (i) BCS requires manual setting or grid search for hyperparameters (e.g., shape parameter of the sparse prior, noise variance), increasing computational load and potentially causing accuracy degradation due to parameter mismatch. VBCS, conversely, adaptively learns all hyperparameters during the optimization of the Kullback–Leibler (KL) divergence. The values of hyperparameters are completely data-driven, which are more consistent with the sparse characteristics and noise levels of real sound fields. Under sparse and weakly coherent conditions, the accuracy gain from this adaptation far outweighs the error introduced by the mean-field approximation. (ii) VBCS combines Gamma priors with variational optimization to dynamically constrain the sparsity of equivalent source coefficients. Specifically, relaxing constraints on non-zero coefficients to preserve information while strengthening constraints on zero coefficients to suppress noise. In contrast, the sparse prior in BCS is more rigid, exhibiting weak adaptability to complex sound fields and being prone to issues such as “over-constraining valid coefficients” or “under-constraining noise”. (iii) Owing to its high computational speed, VBCS can support more rounds of iterative optimization or adopt refined parameter update strategies such as coordinate ascent variational inference under the same computational resources, thereby converging closer to the global optimum. In contrast, BCS incurs high computational complexity and often compromises by reducing the number of iterations, for instance by early termination, so its final solution may become trapped in a local suboptimum, thus widening the accuracy gap with VBCS.

To address the aforementioned limitations, future research could explore the following directions: (i) Introduce structured variational distributions such as full-covariance Gaussian or hierarchical variational models to replace the mean-field assumption, capture statistical correlations among equivalent source coefficients, improve posterior uncertainty estimation, and explore lightweight inference strategies that balance computational complexity. (ii) Integrate hybrid prior models tailored separately for sparse and continuous sources, constructing a variational inference framework capable of adaptive switching to expand applicable scenarios. (iii) Incorporate deep neural networks to learn the structure of the sensing matrix, further optimizing constraint strategies on the solution space under extremely sparse sampling conditions, thereby enhancing algorithm robustness.

## 6. Conclusions

This paper presents a near-field acoustic holography method based on variational Bayesian compressive sensing for sound field reconstruction. The method first acquires sound pressure signals via a hologram plane measurement array and then establishes a sparse representation model of the sound field based on the equivalent source method, thereby transforming the reconstruction problem into a sparse coefficient recovery task under a Bayesian posterior inference framework. To implement this framework, a variational Bayesian inference approach integrated with a real-valued transformation strategy is introduced, assigning Gaussian distributions to the sparse coefficients and Gamma conjugate priors to their variances and noise precision. To address the computational intractability of direct posterior calculation, a mean-field variational inference approach is adopted. By minimizing the Kullback–Leibler divergence between the posterior distribution and its variational approximation, the posterior inference task is converted into the evidence lower bound maximization problem. The method incorporates the mean-field assumption to approximate the joint posterior as a product of independent factors, decomposing the high-dimensional optimization into a series of low-dimensional subproblems. Through iterative updates of variational parameters via the coordinate ascent algorithm, the maximum a posteriori estimate of equivalent source strengths is obtained for sound field reconstruction. While retaining the implicit regularization and overfitting resistance inherent to Bayesian modeling, this method ensures stable algorithmic convergence by monitoring the evidence lower bound, significantly improving computational efficiency while maintaining reconstruction accuracy, thus enhancing the practical feasibility and computational effectiveness of near-field acoustic holography.

Numerical simulations demonstrate that the proposed VBCS algorithm achieves lower reconstruction errors than the conventional BCS and OMP across the entire frequency spectrum. Under limited sampling conditions, VBCS exhibits superior reconstruction accuracy over BCS and significantly outperforms OMP under identical configurations, thereby offering the potential to reduce measurement costs in practical applications. In low signal-to-noise ratio environments, VBCS maintains lower reconstruction errors than both benchmarks, indicating stronger noise resistance. In terms of computational efficiency, VBCS is notably faster and more stable than BCS. While OMP retains an advantage in raw computational speed, it yields the lowest reconstruction accuracy among the three. Experimental results further verify that the VBCS algorithm maintains relative reconstruction errors below 25%, demonstrating its effectiveness and reliability. Even with a limited number of measurement points, its performance remains consistently superior to both conventional BCS and OMP. Crucially, VBCS decomposes the complex joint posterior probability through the mean-field approximation, effectively breaking down the high-dimensional optimization problem into manageable low-dimensional subproblems. This decomposition leads to reduced computation time while maintaining high accuracy. In summary, the VBCS algorithm surpasses BCS and OMP in both reconstruction accuracy and computational efficiency, and provides a robust, practical solution for real-time, high-accuracy sound field reconstruction.

In future work we will extend the research along three avenues: embedding systematic numerical-stability schemes to tackle the severely ill-posed high-frequency reconstruction problem; replacing the mean-field approximation with structured variational distributions to improve probabilistic modeling, interpretability and generalization under sparsity or noise; and fusing deep neural networks with the physics-based model to yield a data-driven yet interpretable hybrid framework capable of capturing complex nonlinear relationships.

## Figures and Tables

**Table 1:** Technical specifications of measurement hardware.

Hardware	Model	Manufacturer
Microphones	46AE	GRAS Sound and Vibration (Skovlytoften 33, 2840 Holte, Denmark)
Power Amplifier	2716C	Brüel and Kjær (Teknikerbyen 28, DK-2830 Virum, Denmark)
Electrodynamic Shaker	4825	Brüel and Kjær (Teknikerbyen 28, DK-2830 Virum, Denmark)
Data Acquisition (DAQ) System	SCM205	LMS (Interleuvenlaan 68, 3001 Leuven, Belgium)

## Data Availability

The original contributions presented in this study are included in the article. Further inquiries can be directed to the corresponding author.
